# New Results on Radioactive Mixture Identification and Relative Count Contribution Estimation

**DOI:** 10.3390/s21124155

**Published:** 2021-06-17

**Authors:** Bulent Ayhan, Chiman Kwan

**Affiliations:** Applied Research LLC, Rockville, MD 20850, USA; bulentayhan@gmail.com

**Keywords:** nuclear isotopes, mixtures, remote detection, deep learning, GADRAS

## Abstract

Detecting nuclear materials in mixtures is challenging due to low concentration, environmental factors, sensor noise, source-detector distance variations, and others. This paper presents new results on nuclear material identification and relative count contribution (also known as mixing ratio) estimation for mixtures of materials in which there are multiple isotopes present. Conventional and deep-learning-based machine learning algorithms were compared. Realistic simulated data using Gamma Detector Response and Analysis Software (GADRAS) were used in our comparative studies. It was observed that a deep learning approach is highly promising.

## 1. Introduction

Illegal use of nuclear materials can cause social unrest and dangers to human lives. It is critical to stop the smuggling of nuclear materials at customs or border checkpoints. Although there are devices such as Sodium Iodide (NaI) and other detectors, they may not function well if the material concentration is low or there are several isotopes mixed together. In recent years, there have been new developments in machine learning/deep learning that have great potential to enhance the detection and classification of nuclear materials with low concentration or mixtures [1,2,3,4]. However, it is still challenging for several reasons. First, machine learning (ML) requires a lot of training data. Fortunately, there are software simulation tools, such as GADRAS [5], that can be used to generate training data. Second, the spectral data collected by the detectors normally contain background noise and interferences. This necessitates robust algorithms for material classification. Third, there may be scenarios where multiple nuclear materials may be present, making the spectral signatures more complicated. Some spectral unmixing may be needed in order to correctly classify the different nuclear materials.

Radiation spectrum analysis has been traditionally done by looking at certain regions of interest (ROI) in the gamma ray spectrum [6,7,8,9]. One drawback of the aforementioned approaches is that they may not perform well when ROIs overlap significantly with large libraries of radio-isotopes. Recently, researchers have taken the entire spectrum into account for isotope identification [10,11]. One key benefit is that the Compton continuum can be taken into account and the entire spectrum is shown to allow for some tolerance to gain shift.

To analyze spectral signatures from a mixture of materials, there are some additional methods that are popular in various fields. Non-negativity Constrained Least Square (NCLS) has been used in chemical agent detection [12]. Partial Least Square (PLS) has been used in rock composition analysis in Laser Induced Breakdown Spectroscopy (LIBS) [13] onboard the Mars rover Curiosity. Deep Belief Network (DBN) has been used in hyperspectral image classification [14]. Linear regression (LR) and Random Forest Regression (RFR) [15] are also conventional machine learning tools that can be used for unmixing analysis.

Deep learning has made significant progress since the seminal work by Hinton’s group in 2012 [16]. After that, deep learning has been widely used in many applications such as target detection and classification [17,18], stock market forecasting [19], land cover classification [20], image enhancement [21], and many more [22].

In this study, we propose to apply both conventional machine learning and recent deep learning approaches to detecting and classifying nuclear materials in mixtures. We mainly focus on mixtures containing multiple nuclear materials. We examined realistic gamma ray spectral data to test our identification and mixing ratio estimation methods. In particular, we used GADRAS to generate realistic spectral data for comparing different algorithms. We then proceeded with two additional investigations. First, we performed relative count contribution (mixing ratio) estimation performance analyses for high and low mixing ratio mixture datasets. Second, we performed robustness analysis on the mixing ratio estimation methods when unknown number of source mixtures is present and also when the source-to-detector distance varies. Third, we studied the use of using foreground spectra only for mixing ratio estimation.

Our contributions are two-fold. First, we thoroughly compare conventional and deep learning algorithms in the context of material detection in mixtures. Second, we demonstrated that a deep learning algorithm has great potential in mixing ratio estimation in mixtures using realistic data generated by GADRAS.

This paper is organized as follows. Section 2 briefly reviews the six algorithms for spectral analysis and relative count contribution/mixing ratio estimation. Section 3 summarizes how the data were generated using GADRAS. Section 4 presents unmixing results using realistic simulated data generated from GADRAS. Finally, some concluding remarks and future directions are mentioned in Section 5.

## 2. Radioactive Mixture Identification and Relative Count Contribution Estimation Algorithms

In this section, we provide brief technical information about the six relative count contribution or mixing ratio estimation methods that were applied in a number of different analyses in this work. One is a signature-based method, three of them are machine-learning-based methods, and two are deep learning methods.

### 2.1. Non-Negativity Constrained Least Squares (NCLS)

A non-machine learning (ML) approach can be useful to handle some unexpected situations such as multiple nuclear materials where the spectrum will be mixed. If known spectral signatures of individual materials are available, then we can apply a technique known as NCLS to unmix the spectrum and classify the materials. We have used NCLS for chemical agent detection [12] before and observed good performance.

The NCLS approach is related to the following optimization problem,

(1) $$\mathrm{Minimize}\;\mathrm{LSE}={\left(M\alpha -r\right)}^{T}\left(M\alpha -r\right)\;\mathrm{subject}\;\mathrm{to}\;\alpha_{j}\geq 0$$

where LSE is the least squares error used as a criterion for optimality, and $\alpha_{j}\geq 0$ represents the non-negativity constraint for 1≤j≤p. In NCLS, suppose the spectra of which its composition estimate is aimed to be found is denoted by **r**, and the gamma ray signature library is denoted by **M**. NCLS aims to estimate **α** (concentration estimates) from **r** (gamma ray spectra of the radioactive mixture sample) and **M** (gamma ray signature library). In order to use the Lagrange multiplier method, a *p*-dimensional unknown positive constraint vector $c={\left[c_{1},c_{2},\ldots ,c_{p}\right]}^{T}$ with $c_{j}\geq 0$ is introduced, where 1≤j≤p and the Lagrange multiplier vector is $\lambda ={\left[\lambda_{1},\lambda_{2},\ldots ,\lambda_{p}\right]}^{T}$. A Lagrangian, J(α), by means of c is formed as

(2) $$J(\alpha )=(1/2){\left(M\alpha -r\right)}^{T}\left(M\alpha -r\right)+\lambda^{T}(\alpha -c)$$

subject to the constraint α=c. Differentiating J(α) with respect to α yields

(3) $${\frac{\partial J(\alpha )}{\partial \alpha }|}_{{\hat{\alpha }}_{\mathrm{NCLS}}}=0=M^{T}M{\hat{\alpha }}_{\mathrm{NCLS}}-M^{T}r+\lambda$$

Equation (1) results in the following two iterative equations

(4) $${\hat{\;\alpha }}_{\mathrm{NCLS}}={\left({Μ}^{T}Μ\right)}^{-1}{Μ}^{T}r-{\left({Μ}^{T}Μ\right)}^{-1}\lambda$$

(5) $${\hat{\alpha }}_{\mathrm{LS}}={\left(M^{T}M\right)}^{-1}M^{T}r$$

Equations (4) and (5) can be used iteratively to solve the optimal solution ${\hat{\alpha }}_{\mathrm{NCLS}}$. Details of the NCLS can be found in [12].

### 2.2. Partial Least Squares (PLS)

Suppose a process is modeled by

(6) Y=XB+E

where $X\in {ℜ}^{N\times m}$ and $Y\in {ℜ}^{N\times l}$ are the input and output data matrices, and $B\in {ℜ}^{m\times l}$ is a parameter matrix. Suppose X is defined as

(7) $$X=\left[\begin{array}{l} {x_{1}}^{\prime }\\ {x_{2}}^{\prime }\\ \;\vdots \\ {x_{N}}^{\prime } \end{array}\right]\in {ℜ}^{N\times m}$$

where $x_{i}\in {ℜ}^{m}$ is the $i^{th}$ observation of the inputs [13]. In PLS, suppose the gamma ray spectra are denoted by **X** and the radioactive material compositions of **X** are denoted by **Y**. The PLS model is then based on predicting **Y** from **X**, where **Y** = **XB**; that is, PLS estimates **B**. We have applied PLS in LIBS data analysis for rock composition determination before [13].

### 2.3. Deep Belief Network (DBN)

Restricted Boltzmann Machines (RBM), which are generative undirected graphical models, make up the architecture of the Deep Belief Networks (DBNs) [23]. DBNs are formed by stacking RBMs and training the units of the DBN architecture with stochastic gradient descent techniques [23]. In an RBM, the lower layer is called the visible layer and the top layer is the hidden layer. The units in the two layers can be interpreted as stochastic binary variables [23]. These units are connected to each other via undirected weights. An RBM can learn a probability distribution over its set of inputs [24]. Thus, a trained RBM model can generate data like the training data. Training RBM consists of maximizing the product of probabilities assigned to training dataset. RBMs can be also configured to be used for classification problems [25].

In training of a DBN which consists of multiple stacked layers of RBMs, the first RBM is trained, then the second RBM is trained using the first RBM’s hidden layer as the second RBM’s visible layer [23], and this process repeats itself for all RBM layers. The last layer of DBN, which is the label layer, can be any standard classifier [23]. In this work though, we used DBN for a regression problem. DBN is used as one of the benchmark methods in this work since it consists of stacking learning units in its architecture for deep learning. It has also been noted that due to its use of RBMs, DBNs can mitigate the impact of noise that can be present in the training data [23].

Deep Belief Network (DBN) [14] used in this work is a three-level DBN architecture with sigmoid activation function, which can be seen in the following. The first two levels of DBN architecture consist of RBMs.

(a) Level-1 (RBM with 50 hidden units): 1000 epoch training
(b) Level-2 (RBM with 50 × 50 hidden units): 1000 epoch training
(c) Level-3 (connection to y (output) with neural network (NN)): 30,000 epoch training

### 2.4. Dense Deep Learning for Regression

Dense layer, also known as fully connected (FC) layer, is a neural network layer in which a neuron in this layer receives input from all neurons in the previous layer. The dense layer can be considered as performing a matrix vector dot product where matrix is the weights matrix and vector is the input tensor [26]. The bias vector is added to the result of the dot product followed by applying an activation function to introduce nonlinearity in the network. By stacking dense layers, deep learning architectures can be formed which can be used for classification and regression problems. Depending on the problem type, choice of loss function and optimization method in model training becomes important. The mixing ratio estimation performance of a dense deep learning model for multi-input multi-output regression is examined in this investigation. We have selected a deep learning architecture with dense layers since their effectiveness was demonstrated in classification and regression problems.

The dense deep learning model is designed using Keras’s sequential model [15]. The sequential model contains three Dense layers with Rectified Linear Unit (ReLU) activations. For optimization, Adam optimizer [27] is used. After an optimal hyperparameter search, the number of neurons in the first layer is set to 800. The number of neurons in the second layer is set to 256. The number of neurons in the third layer is set to 13 which is the same number of radioactive isotope materials. The dense deep learning architecture is depicted in Table 1. We will call this model “Deep Regression (DR)” throughout the rest of this paper when presenting the conducted analyses.

### 2.5. Linear Regression and Random Forest Regression Algorithms

We also used linear regression (LR) and random forest regression (RFR) algorithms in the Keras library [15] in our investigations. These are considered as classical benchmark machine-learning-based methods for regression problems, and they are included in this work for comparison purposes with the deep-learning-based methods.

Since linear regression (LR) [28] is a well-established method, we will not provide technical details. Random forest regression (RFR) [29] is an ensemble method in which multiple decision trees (DTs) are constructed. DT is a non-parametric supervised learning method that predicts the value of a target by learning decision rules and these rules are extracted from the data features [30]. For an unseen test data sample, the decisions from each of the DTs in RFR are averaged to finalize the output decision [30]. In this work, after a number of trial and errors to find the best performance, the number of DTs in RFR is set to 100. RFR due to its nonlinearity can perform better than linear methods. However, it has some disadvantages such as overfitting and the need to search for an optimal number of DTs in the model training.

## 3. Radioactive Mixture Data Generation Using GADRAS

GADRAS is a powerful simulation tool that can generate realistic gamma ray spectra under various conditions. We familiarized ourselves with GADRAS [5] software so that we can emulate gamma ray spectra of individual radioactive materials and also synthesize mixtures of these radioactive materials at different relative count contributions. We then performed radioactive material mixture simulation using GADRAS’ Inject tool. We used GADRAS to generate mixtures of different radioactive materials with different relative count contributions. We also use the term ‘relative count contribution’ for the mixing ratio.

### 3.1. Detector Settings in GADRAS Simulation

**Figure 1:**
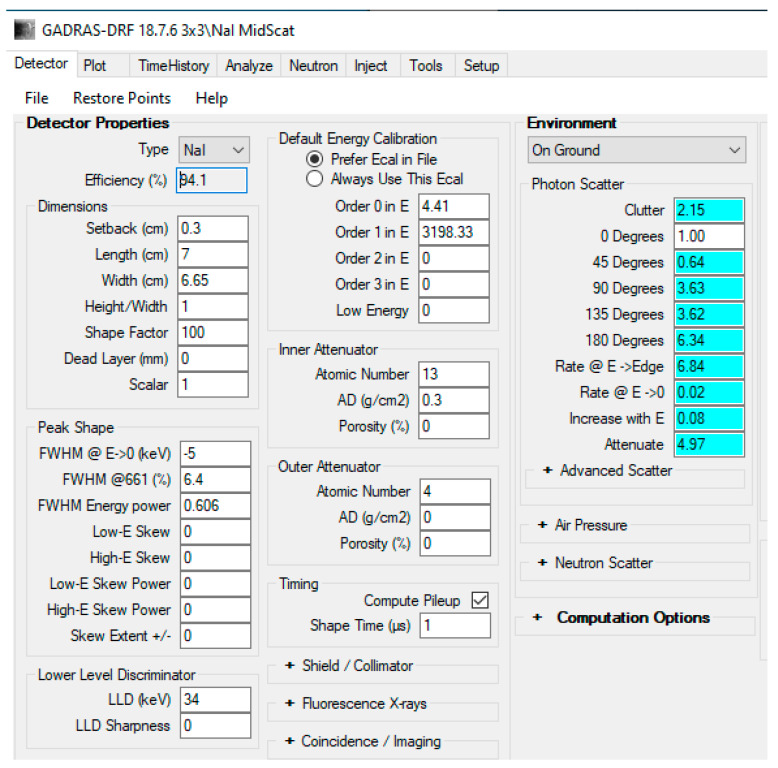
NaI Detector parameters used in the GADRAS simulation.

In the conducted investigations, NaI detector was used in GADRAS [5] to simulate the detector responses to the radioactive materials and their mixtures in gamma ray spectra. The height for the detector was set to 56 cm (H = 56 cm) and the distance of the detector to the radioactive materials was set to 122 cm (@ 122 cm, H = 56 cm). The complete Sodium Iodide (NaI) detector parameters used in the simulation can be seen from the GADRAS graphical user interface (GUI) screenshot for the Detector tab in Figure 1.

### 3.2. Radioactive Materials Used in the Individual Source-Only Simulation with GADRAS’ Inject Tool

According to [31], most of the nuclear trafficking consists of a certain group of radioactive materials. The second column of Table 2 shows 13 of these radioactive materials. When we checked GADRAS, we saw that these nuclear materials exist in GADRAS’s isotopes library. Using this library, we generated individual gamma ray signatures of these 13 radioactive materials with respect to the NaI detector (122 cm @ H = 56 cm) in GADRAS. Using these simulated signatures, we created a signature library to identify materials and quantify their relative count contributions using spectral unmixing-based methods which require a signature library in advance like the NCLS method. The activity units for these 13 radioactive materials are in Curies (Ci) and the base activity units for each radioactive material when forming the signature library are selected such that the detector responses to these materials have similar amplitudes. The selected units for these materials can be seen in Table 2.

**Figure 2:**
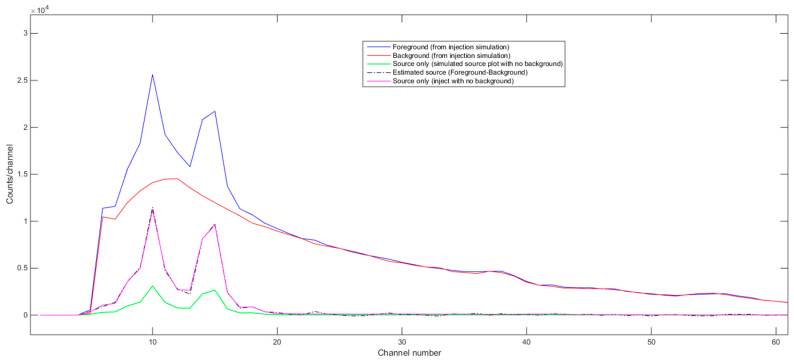
Estimated source spectra (foreground-background) using the inject tool of GADRAS with background, using Plot tool alone and using the Inject tool with no background and no Poisson statistics for ^238^U, 10 uCi source with NaI detector @ 122 cm, H = 56 cm.

**Figure 3:**
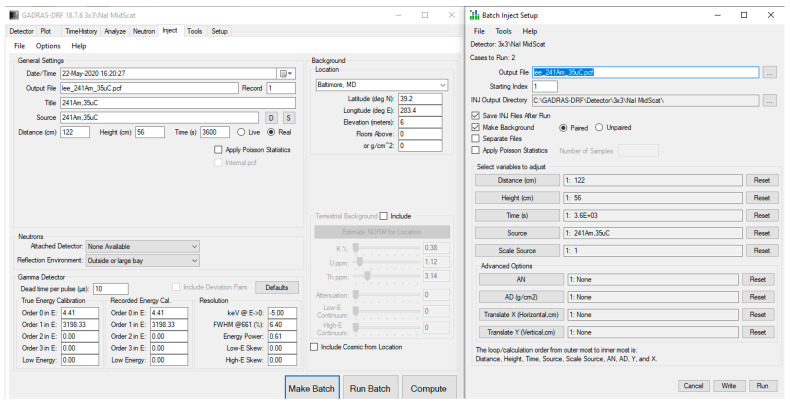
GADRAS Inject tool settings for generating individual radioactive source-only spectra with NaI detector @ 122 cm, H = 56 cm.

**Figure 4:**
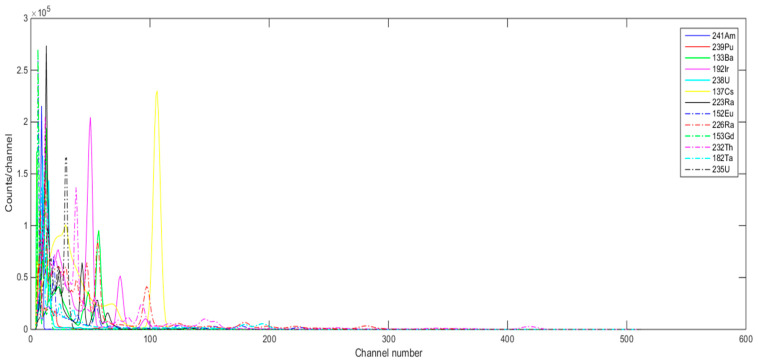
Gamma ray signatures of the individual radioactive materials generated with GADRAS (NaI detector @ 122 cm, H = 56 cm).

To emulate the individual material gamma ray spectra with the NaI detector when forming the signature library, we considered three different ways in GADRAS. These are: (1) using GADRAS’s Inject tool with foreground and background (with Poisson statistics) and then subtracting background from foreground, (2) using GADRAS’s Plot tool that plots just the detector response to the isotope in gamma ray spectrum, and (3) using GADRAS’s Inject tool with no background and no Poisson statistics with NaI detector @ 122 cm, H = 56 cm, meaning that a source-to-detector distance is 122 cm and the height of the detector is 56 cm above ground. Figure 2 shows an estimated source spectrum (foreground-background) for one of the radioactive materials (^238^U, 10 uCi) using these three different ways. As can be seen from Figure 2, even though the background-subtracted spectrum (first way) is almost the same in value and shape to the individual material spectrum using GADRAS’s Inject tool without background simulation (third way), the spectrum using GADRAS’s Plot tool generated a spectrum similar in shape but with a very different amplitude scale. Because we found this to be confusing and not very intuitive, we communicated with the GADRAS team in Sandia Labs regarding this and shared our observations. We received a quick response from them saying that this issue would be fixed in the next release of GADRAS. For this reason, for the signature library, we generated the pure individual source-only spectra of these 13 radioactive materials, using the third way which consists of using GADRAS’ Inject tool with no background and no Poisson statistics for NaI detector @ 122 cm, H = 56 cm. These individual gamma ray spectra of 13 materials are used when forming the signature library in NCLS. Figure 3 shows the batch inject setup screenshot in GADRAS for one of the 13 radioactive sources. Figure 4 shows the resultant gamma-ray spectrum signatures of the 13 radioactive materials in the signature library for NCLS with the activity units listed in the third column of Table 2.

### 3.3. Radioactive Material Mixture Simulation Using GADRAS’ Inject Tool

For radioactive mixture data, we generated all combinations from 13 radioactive materials. In particular, there are 78 combinations in two-source case, 286 combinations in the three-source case, 715 combinations in the four-source case, and 1287 combinations in five-source case.

For the relative count contributions of materials that are present in the emulated mixtures, two different relative count contribution or mixing ratio ranges were considered. The first mixing ratio range corresponds to a range between 0.25 and 3. This range can be considered for the scenario where there is abundant radioactive material such that the detector response for the mixture is significantly bigger in amplitude than the detector’s response for background. The mixing ratio of the material in the emulated mixture is determined randomly within this mixing ratio range for that radioactive material. As an example, a mixing ratio value of 1.2 for ^137^Cs indicates that the curie unit of ^137^Cs is 180 uCi in the mixture since the base curie unit for ^137^Cs was 150 uCi and 1.2 mixing ratio unit of base ^137^Cs, 150 uCi corresponds to 180 uCi (1.2 × 150 uCi = 180 uCi).

The second mixing ratio range corresponds to a range between 10^−3^ and 10^−1^. This mixing ratio range can be thought for the scenarios where the detector responses for the radioactive mixture and background have similar amplitude scales. It is worth mentioning that when the mixing ratio range is selected lower than this range, the background-subtracted spectra start to have oscillating waveform shapes around 0, which are not reliable for applying spectral unmixing methods. For this reason, we did not consider mixing ratio ranges lower than this in the investigations.

For each two-source mixture combination, 55 mixtures of this same material combination with different mixing ratios are generated which makes a total of 4290 two-source mixtures (78 × 55). For each three-source mixture combination, 15 mixtures of the same mixture combination with different mixing ratios are generated which makes a total of 4290 three-source mixtures (286 × 15). For each four-source mixture combination, six mixtures of the same mixture combination with different mixing ratios are generated which also makes a total of 4290 four-source mixtures (715 × 6). Finally, for each five-source mixture combination, three mixtures of the same mixture combination with different mixing ratios are generated which makes a total of 3861 three-source mixtures (1287 × 3).

**Figure 5:**
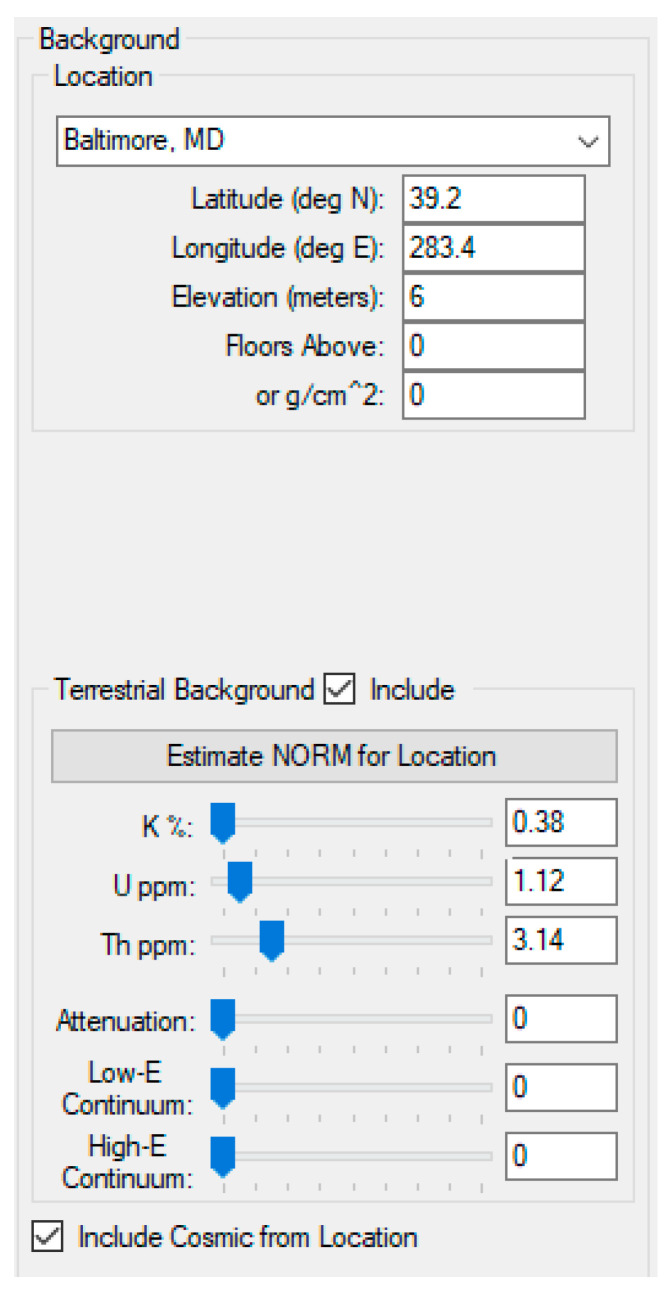
GADRAS Inject tool settings for generating background spectrum with NaI detector @ 122 cm, H = 56 cm (Baltimore, MD location is used, and terrestrial and cosmic are included).

The GADRAS inject tool is used to generate the detector responses for the mixtures in gamma ray spectrum. This tool emulates both the measured foreground and background spectra. The mixture spectrum is then obtained by subtracting the background spectrum from the foreground spectrum. For background spectrum, in GADRAS, the location is set to Baltimore, Maryland (MD). Terrestrial and cosmic from location are also included in background spectrum simulation. The parameter settings for background spectrum simulation used in GADRAS can be seen in Figure 5.

**Figure 6:**
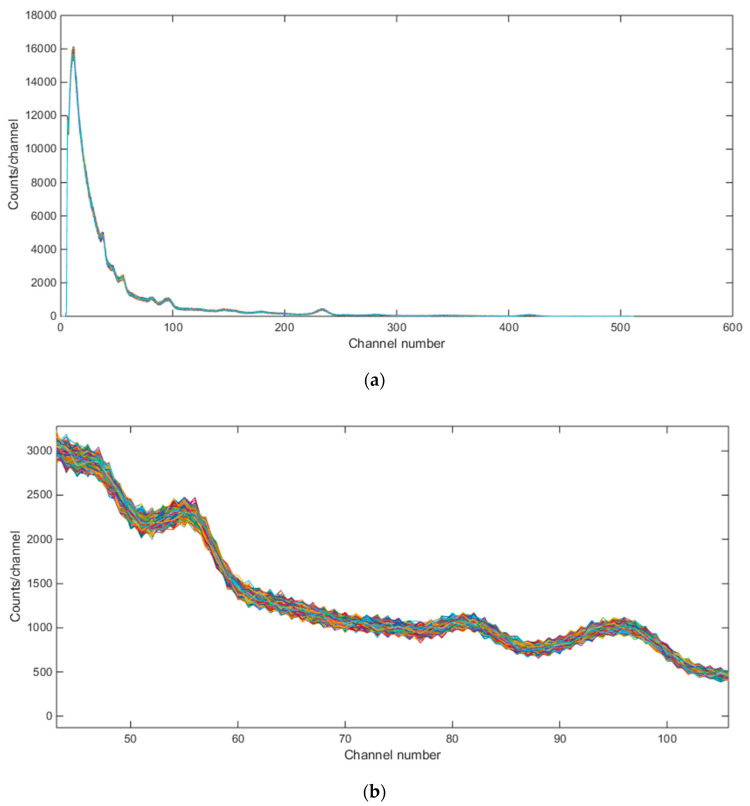
Emulated background gamma ray spectra (for Baltimore, MD) for 4290 separate simulations. (**a**) whole spectrum; (**b**) zoomed version.

It is worth mentioning that for each emulated mixture foreground gamma ray spectrum, we selected ‘paired make background’ option in the Inject tool which also emulated a paired background gamma ray spectrum using the background settings in Figure 5. Since we needed to emulate thousands of different-source mixture combinations, we created inject text files for each mixture combination using an automated Matlab script and then used GADRAS’s “Run Batch” command which used these inject text files in a loop to synthesize these spectra. As an example, the background spectra for the 4290 three-source mixtures can be seen in Figure 6. When zoomed to these background spectra as can be seen in Figure 6b, the variation in the background spectra can be seen fine. However, overall, the variations in the emulated background spectra with GADRAS are not found to be large as was initially that thought they would be.

## 4. Radioactive Mixture Identification and Mixing Ratio Estimation Results Using GADRAS Generated Data

In Section 4.1, we will summarize the results from the adaptation of a deep learning-based regression method for mixing ratio estimation (quantification) of multi-source mixtures. This section will also contain results from three other methods for comparison purposes with the deep-learning-based method. The dense deep learning model was applied to previously generated GADRAS datasets (“high-mixing-rate” and “low-mixing-rate” two-source, three-source, four-source and five-source datasets) which consist of different combinations of 13 isotopes. The detector was NaI. These datasets can be considered as highly homogeneous datasets since the detector parameters such as source height, detector-to-source distances, shielding and shielding density were set to fixed values when generating the mixture spectrum data in these datasets. Deep regression method is found to perform better than other three methods for all considered mixture cases of the high-mixing-rate mixture dataset. Similarly, it is also found to perform better than other methods for all considered mixture cases of the low- mixing-rate mixture dataset with the exception of the five-source mixture dataset. Deep regression method is found to be robust when a higher number of source mixtures are used for training and when the test dataset consists of smaller number of source mixtures. However, for the opposite case, PLS is observed to be more robust than the deep regression method. The conducted investigations above used background-subtracted foreground spectra (source) for mixing-rate estimation. Considering there could be cases where background measurements are not available, the previously applied methods and the low-mixing-rate mixture dataset are used for mixing-rate estimation with foreground spectra only. The results showed that NCLS method performs poorly when foreground spectra are used. Deep regression method, on the other hand, is found to perform better than other methods including the five-source mixture case.

In Section 4.2, we will summarize our investigations on how the mixing-rate estimation methods would perform when the detector-to-source distance varies; namely, when the dataset of interest, is not homogeneous. We considered low-mixing-rate two-source mixture dataset and using GADRAS we generated training data for five different source-detector distances, which are 72, 122, 172, 222 and 272 cm. A total of 13 isotopes are used when forming the mixture spectrum data like before. When source spectra and foreground spectra are used, deep regression method is found to perform better than others followed by the RFR method. This investigation also showed that when the test dataset mixtures have a detector-to-source distance that is different than the one which NCLS signature library is formed of, NCLS performed poorly.

### 4.1. Relative Count Contribution (Mixing Ratio) Estimation Performance Analyses for High and Low Mixing Ratio Mixture Datasets

#### 4.1.1. High Mixing Ratio Mixture Dataset Results

**Figure 7:**
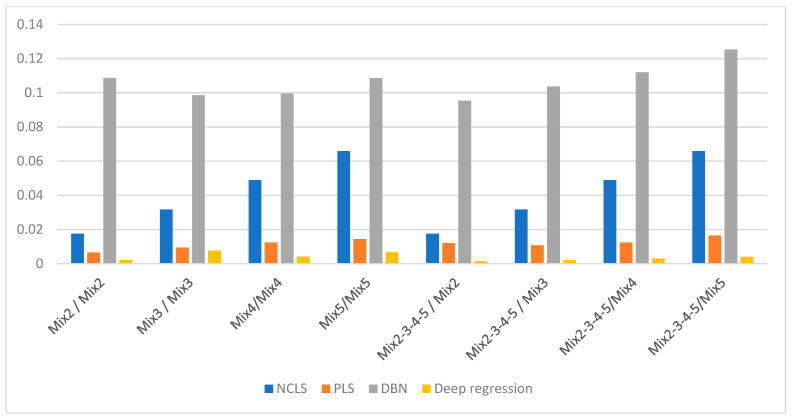
Average RMSE comparison using bar plots for “high-mixing-rate mixture” dataset with respect to case (Mixing-rate range used for synthesizing mixtures is: [0.25 3.00], Min: 0.25 Max: 3.00).

**Figure 8:**
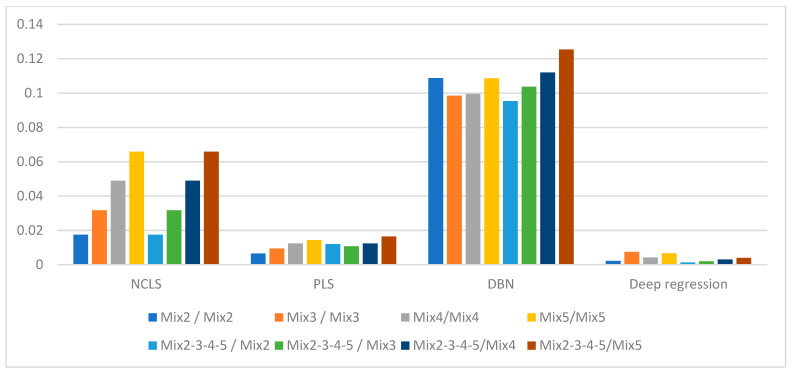
Average RMSE comparison using bar plots for “high-mixing-rate mixture” dataset with respect to method (Mixing-rate range used for synthesizing mixtures is: [0.25 3.00], Min: 0.25 Max: 3.00).

**Figure 9:**
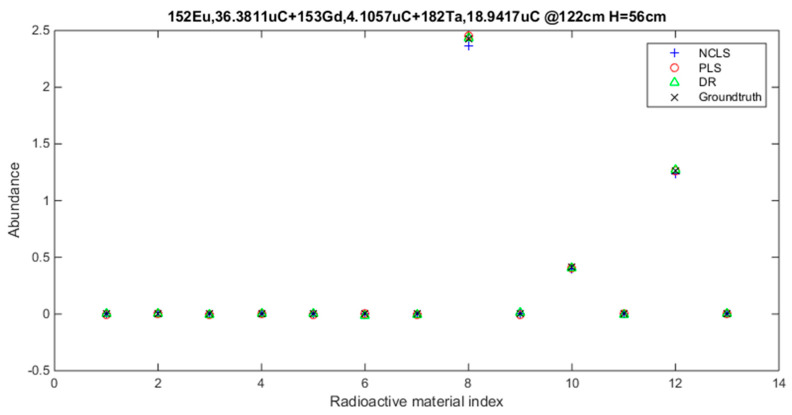
Three-source mixture mixing-rate estimation example (source = background − subtracted foreground) from high mixing-rate dataset, (RMSE values: NCLS: 0.0181, PLS: 0.0090, DR: 0.0066).

Table 3 shows the average root mean square error (RMSE) results for “high-mixing-rate mixture” dataset which represents the high signal-to-background ratio case. When forming this dataset, the mixing-rate range used for synthesizing mixtures was set to: [0.25 3.00], Min: 0.25 Max: 3.00. It is worth mentioning that when the mixing ratio estimation methods are applied to this dataset, background-subtracted foreground spectrum (which is equivalent to source spectrum) are used for training models and for testing with the assumption that one will have the corresponding background spectrum measurement in hand. Figure 7 shows the average RMSE plots in bar chart with respect to mixture case and Figure 8 shows the same results in bar chart with respect to applied mixing ratio estimation method. It can be seen from Table 3 and the two bar chart plots that deep regression (DR) method performs better than other methods for all considered mixture cases of the high mixing-rate mixture dataset. Figure 9 shows an example for three-source mixture mixing ratio estimation in which source spectrum is used with the mixing-rate estimation methods (source = background-subtracted foreground) in the high-mixing-rate dataset. The resultant RMSE values for the three methods are: NCLS: 0.0181, PLS: 0.0090, DR: 0.0066.

#### 4.1.2. Low-Mixing-Rate Mixture Dataset Results

**Figure 10:**
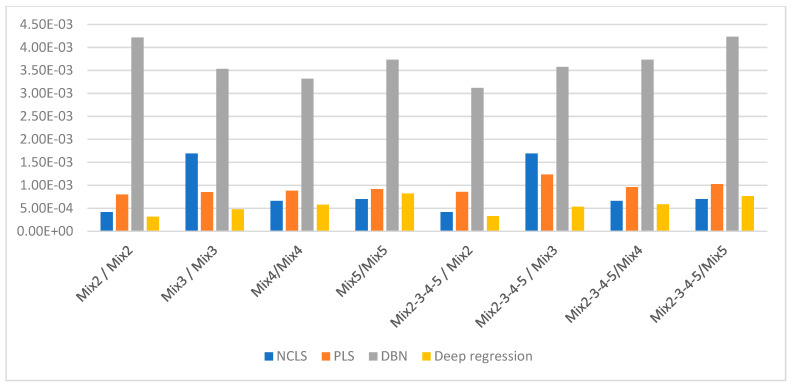
Average RMSE comparison using bar plots for “low-mixing-rate mixture” dataset with respect to case (Mixing-rate range used for synthesizing mixtures is: [10^−3^ 10^−1^], Min: 10^−3^ Max: 10^−1^).

**Figure 11:**
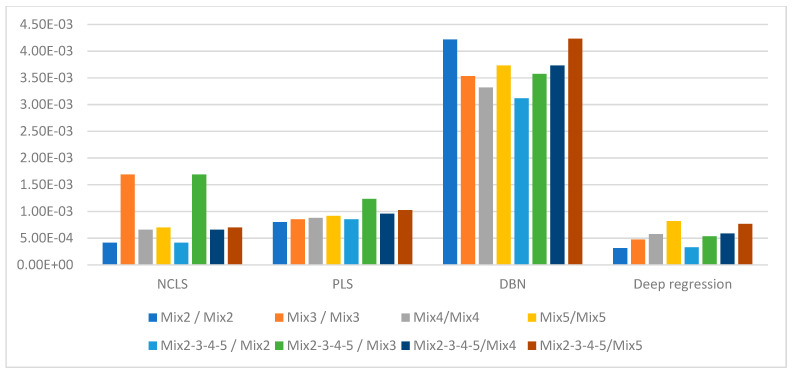
Average RMSE comparison using bar plots for “low-mixing-rate mixture” dataset with respect to method (Mixing-rate range used for synthesizing mixtures is: [10^−3^ 10^−1^], Min: 10^−3^ Max: 10^−1^).

Table 4 shows the average RMSE results for “low-mixing-rate mixture” dataset which represents the low signal-to-background ratio case. When forming this dataset, the mixing-rate range used for synthesizing mixtures was set to: [10^−3^ 10^−1^], Min: 10^−3^ Max: 10^−1^. When the mixing-rate estimation methods are applied to this dataset, background-subtracted foreground spectrum (which is equivalent to source spectrum) are used. However, because Poisson process is applied to both foreground and background spectra with GADRAS during the simulation and because the mixing ratios of mixed sources are set to smaller values, the background and foreground spectra at the end become very close in magnitude. Thus, the use of background-subtracted foreground spectra in low-mixing-rate datasets makes it more challenging for the mixing-rate estimation methods when compared to using mixed sources with high mixing ratios. Figure 10 shows the average RMSE plots in bar chart with respect to mixture case, and Figure 11 shows the same results in bar chart plot with respect to applied mixing-rate estimation method. It can be seen from Table 4 and the two bar chart plots that deep regression method performs better than other methods for all considered mixture cases of the low-mixing-rate mixture dataset with the exception of five-source mixture case.

#### 4.1.3. Robustness Analyses of the Mixing-Rate Estimation Methods to Unknown Number of Source Mixtures in High and Low-Mixing-Rate Mixture Datasets

We examined how robust the applied mixing-rate estimations methods were to new mixture data. With robustness, it is meant how the methods respond when different number source-mixtures were introduced as test data which were not included in model training. Even though NCLS does not require model training, we still included it in the results. DBN is not included in this analysis since its results did not look comparable to other three methods from previous performance analyses. Table 5 and Table 6 correspond to the robustness analysis results for the low and high-mixing-rate mixture datasets. We also note our observations for this investigation as follows:

- For the high mixing-rate dataset, deep regression method is found to be robust when higher number of source mixtures are used in training and when the test dataset consists of smaller number of source mixtures. However, for the opposite case, PLS seems to be more robust than deep regression.
- For the low-mixing-rate dataset, NCLS performed the best among the three. In DR, when lower number of source mixtures are used in training and the test dataset consists of higher number of source mixtures, deep-learning-based regression did not perform well. For the opposite case, deep regression’s performance was better than PLS, but it was still not as good as NCLS.

#### 4.1.4. Using Foreground Spectra (Source + Background) for Mixing-Rate Estimation

**Figure 12:**
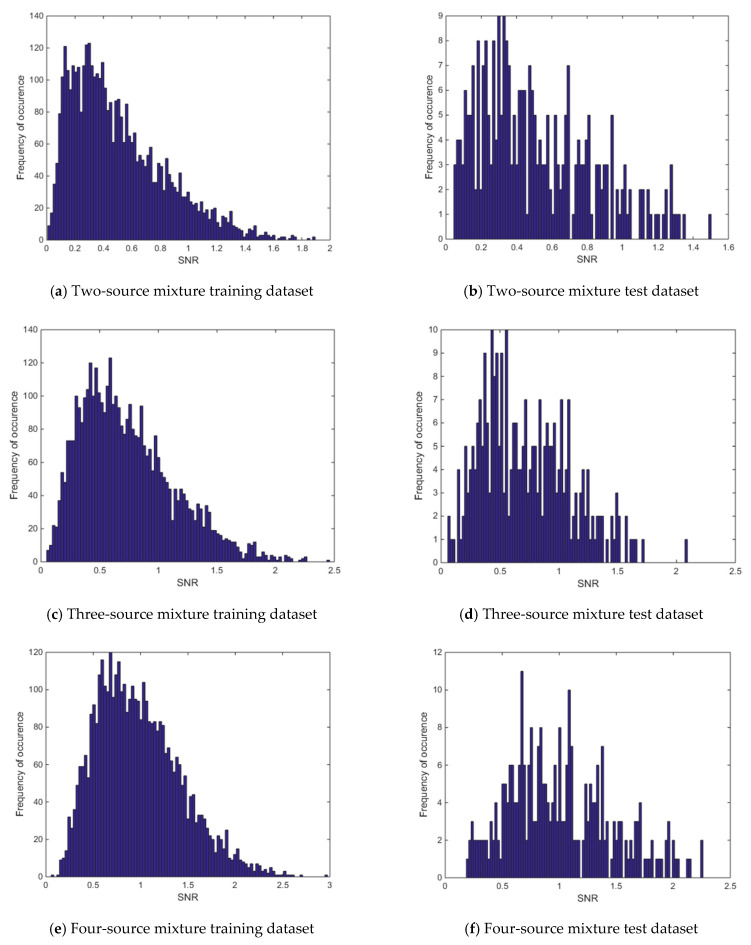

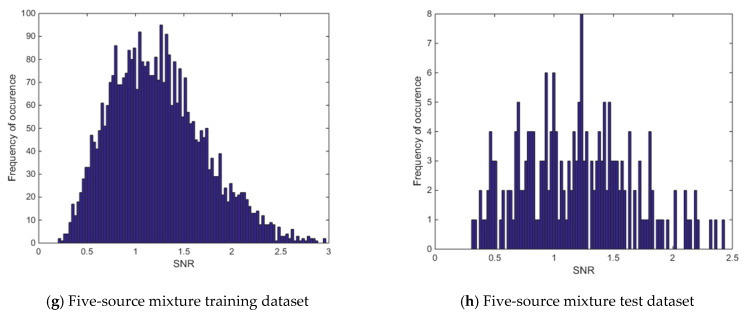
Histogram of signal-to-background ratios for the low-mixing-rate dataset.

In earlier investigations, it is assumed that in addition to foreground gamma ray spectrum measurement, another measurement for the background would also be done. This background spectrum is then subtracted from foreground spectrum and the resultant spectrum, which is an estimate for source, is used for mixing-rate estimation. However, there could be cases where background spectrum is not available and only the foreground spectrum needs to be used for mixing-rate estimation. For this investigation, the previously applied methods with the low-mixing-rate mixture dataset are considered using foreground spectra only. The signal to background ratio for the low-mixing-rate dataset is found to have a range between ~0.1 and ~2.5. The histograms of the signal-to-background ratio values for the low-mixing-rate mixture datasets for both training and test datasets can be seen in Figure 12. The signal-to-background ratios are computed in the following way. Suppose the total number of counts in the foreground spectrum of a multiple-source mixture is *F* and the total number of counts for the corresponding background spectrum of that multiple-source mixture is *B*. The signal-to-background ratio is then computed as:

Signal-to-background ratio = (*F* − *B*)/*B* (8)

**Figure 13:**
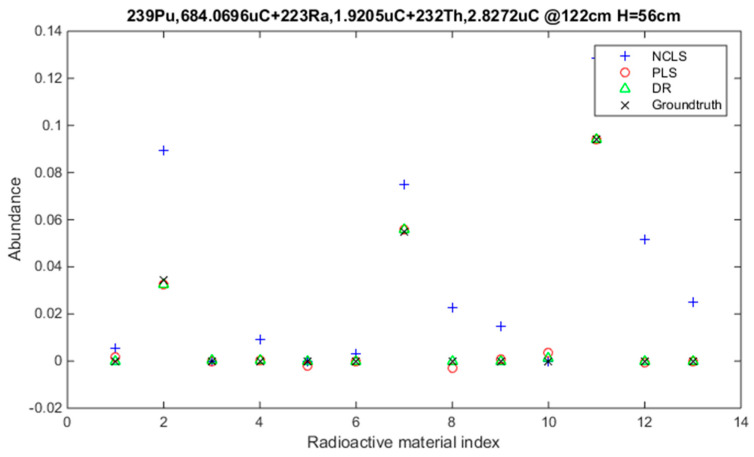
Three-source mixture example from low-mixing-rate dataset when foreground spectrum is used for mixing-rate estimation (RMSE values: NCLS: 0.0260, PLS: 0.0016, DR: 0.0007).

Table 7 shows the average RMSE results when foreground spectra are used for mixing-rate estimation in the “low-mixing-rate mixture” dataset. It can be noticed that NCLS method performs poorly when foreground spectra are used. This is because the signature library for NCLS consists of source-only signatures and when foreground spectra are used for testing, the mixing-rate estimations by NCLS with source-only signature library are not accurate anymore. For all cases, deep regression (DR) method is found to perform better than other methods including the five-source mixture case. Figure 13 shows an example of mixing-rate estimation for a three-source mixture from the low-mixing-rate dataset when foreground spectrum is used for mixing-rate estimation. The RMSE values of the three methods for this example are: NCLS: 0.0260, PLS: 0.0016, DR: 0.0007. From Figure 13, it can be seen that NCLS’s estimates are not very accurate whereas PLS and DR perform very good with DR slightly performing better than PLS.

### 4.2. Simulating Gamma Ray Mixture Spectra for NaI Detector with Various Source Detector Distances and Checking the Robustness of the Mixing-Rate Estimation Methods

**Figure 14:**
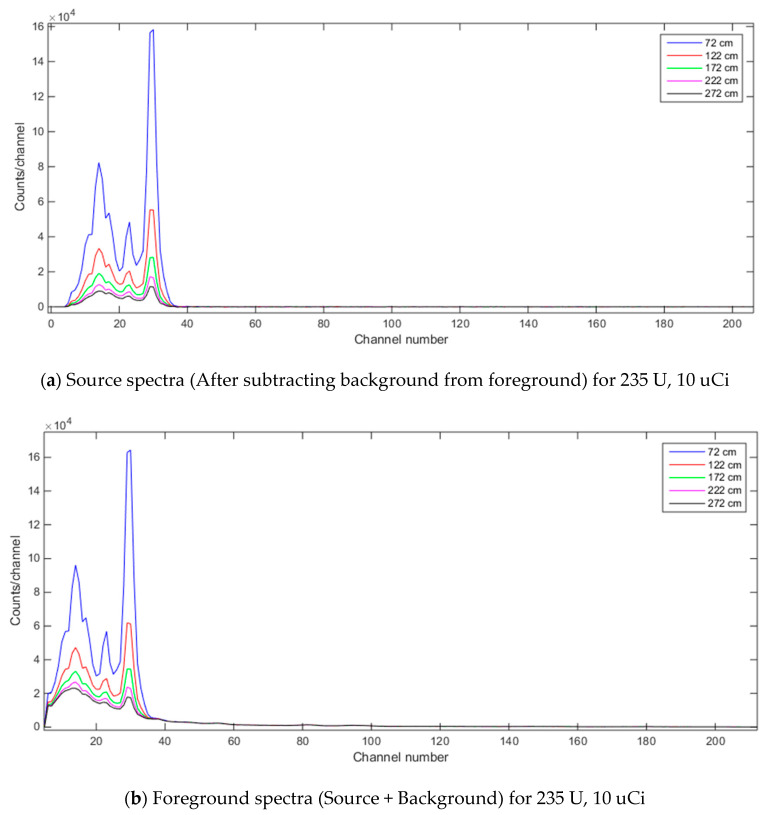
Foreground and source spectra for 235 U, 10 uCi when source-to-detector distance varies (NaI detector where detector-to-ground distance is set to 56 cm and dwell time for detector is 3600 s).

In the previous investigations when forming training and test datasets or when forming signature library (for NCLS method), the multi-source mixture spectra were always formed with a constant detector-to-source distance. In this investigation however, it is assumed that this detector-to-source distance can vary. The objective of this investigation is thus to examine how the mixing-rate estimation methods would perform when the detector-to-source distance varies. We included Linear Regression (LR) and Random Forest Regression (RFR) methods in this analysis to further enhance the scope of comparison with other benchmark methods. The gamma ray spectra (source and foreground spectra) for the ^235^U, 10uCi isotope with the previously used NaI detector (@56 cm detector height) with five different detector-to-source distances can be seen in Figure 14. The source-to-detector distances in this demonstration are 72, 122, 172, 222 and 272 cm. From Figure 14, it can be clearly seen how significantly the spectra change with different source-to-detector distance for the same radioactive isotope. This, in a way, shows the challenges with signature-based mixing-rate estimation methods, such as NCLS, since the signatures highly vary with respect to the detector parameters such as detector-to-source distance or detector height and these signature-based methods would need multiple versions of spectrum signatures for these detector parameter combinations.

**Figure 15:**
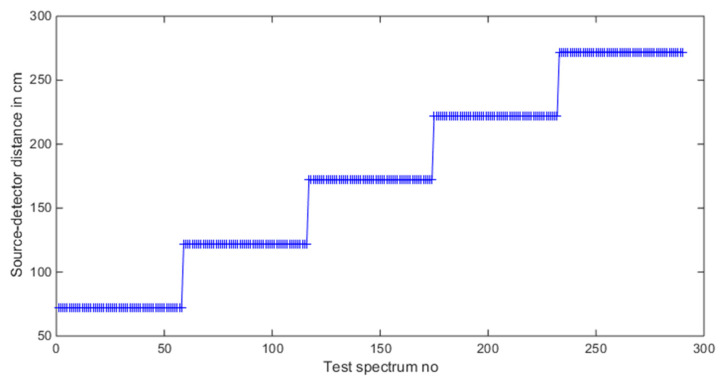
Source-detector distance values in the test dataset.

We considered low-mixing-rate two-source mixtures in this investigation, and using GADRAS, we generated training data for five different source-detector distances which are 72, 122, 172, 222 and 272 cm. A total of 13 radioactive isotopes are used when forming the mixture data like before. As a reminder, the list of the 13 isotopes can be seen in Table 2. For each source-detector distance, 858 two-source mixtures are generated of which their mixing ratios are randomly picked. The mixing-rate range used for assigning mixing ratios when synthesizing two-source mixture is: [10^−3^ 10^−1^], Min: 10^−3^ Max: 10^−1^. Among the 858 spectra for a detector-source distance, 800 of them are used in training set and 58 of them are used for test dataset. Thus, with all five different detector-to-source distance cases, the total number of training dataset becomes 4000 and the total number of test dataset becomes 290. The detector-to-source distances for the test dataset can be seen in Figure 15.

For NCLS signature library, the signatures for the isotopes were obtained when detector-to-source distance was set to 122 cm. For the evaluation, we considered the case in which we assume we have the background spectrum and use the background-subtracted foreground spectrum (equivalent to source spectrum) and also consider the case in which we do not have background spectrum and directly use the foreground spectrum.

**Figure 16:**
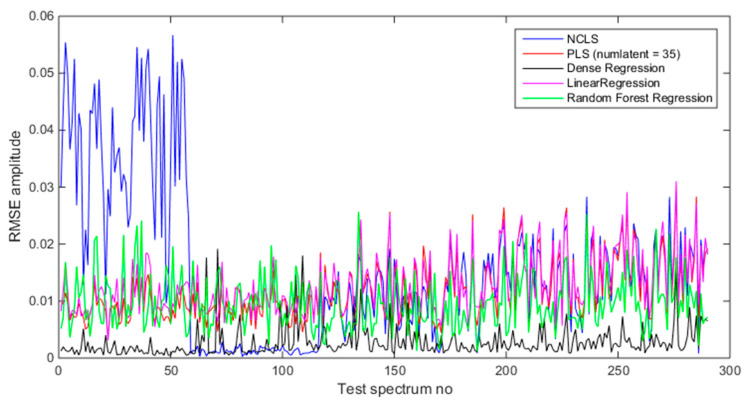
Mixing ratio estimation results when using source-only spectra (foreground − background).

**Figure 17:**
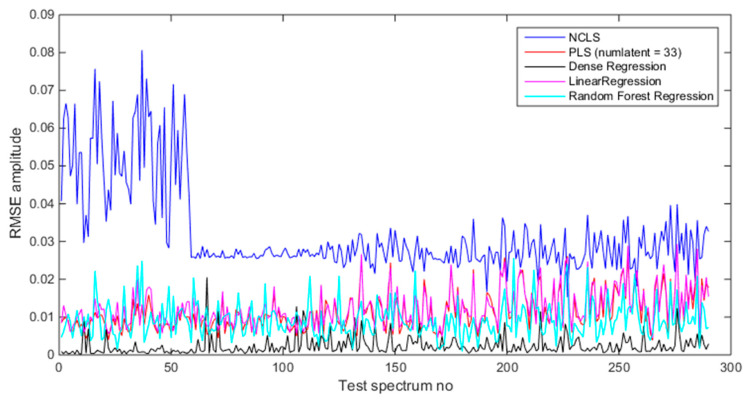
Mixing ratio estimation results when using foreground spectra (source + background).

Table 8 shows the average RMSE values of the test dataset with five methods using source and foreground spectra. In both two cases, deep regression method performs better than others followed by the RFR method. Figure 16 shows the mixing-rate estimation results for the test dataset when using source-only spectra (source = foreground − background). This plot provides more insights about NCLS. It can be seen that when test dataset consists of mixtures where detector-to-source distance is 122 cm, the RMSE values are very small for NCLS and provides RMSE values even as small as deep regression method. However, when the test dataset mixtures are generated with a different detector-to-source distance, NCLS performs poorly. Figure 17 shows the mixing-rate estimation results when using foreground spectra (source + background). In this case, the performance of NCLS becomes even poorer since the NCLS signature library is formed from source-only spectra. Overall, from this investigation we observed that the deep regression method performs very well followed by the RFR method. Among PLS and LR, PLS is found to perform better than LR.

## 5. Conclusions and Future Research

In this paper, we present new results in using conventional and deep-learning-based algorithms for unmixing multiple nuclear materials from mixed spectra. Six algorithms were compared in a number of analyses. It was observed that using realistic data from GADRAS, the deep regression method (DR) yielded very accurate relative count estimation results. One potential research direction is to compare different algorithms using actual spectra

## Figures and Tables

**Table 1:** Dense deep learning model for multi-input multi-output regression (DR).

Model	Layers	Optimizer
Sequential	Layer 1: 800 nodes with ReLu; Layer 2: 256 nodes with ReLu; Layer 3: linear	Loss function: Mean Square Error; Optimizer: Adam

**Table 2:** Radioactive materials and their activity units used in the GADRAS simulation with NaI detector.

Index	Radioactive Material	Unit in Curies (Ci)
1	^241^Am	35 uCi
2	^239^Pu	20,000 uCi
3	^133^Ba	30 uCi
4	^192^Ir	30 uCi
5	^238^U	150 uCi
6	^137^Cs	150 uCi
7	^223^Ra	35 uCi
8	^152^Eu	15 uCi
9	^226^Ra	40 uCi
10	^153^Gd	10 uCi
11	^232^Th	30 uCi
12	^182^Ta	15 uCi
13	^235^U	30 uCi

**Table 3:** Average RMSE results for “high-mixing-rate mixture” dataset (Mixing-rate range used for synthesizing mixtures is: [0.25 3.00], Min: 0.25 Max: 3.00). Bold numbers indicate best performing algorithm.

Training Data	Test Data	NCLS	PLS	DBN	DR
Two-source mixtures	Two-source mixtures	0.0175	0.0065	0.1087	**0.0022**
Three-source mixtures	Three-source mixtures	0.0317	0.0094	0.0985	**0.0075**
Four-source mixtures	Four-source mixtures	0.0489	0.0124	0.0995	**0.0042**
Five-source mixtures	Five-source mixtures	0.0659	0.0143	0.1086	**0.0066**
Merged two-three-four-five-source mixtures	Two-source mixtures	0.0175	0.0120	0.0953	**0.0013**
Merged two-three-four-five-source mixtures	Three-source mixtures	0.0317	0.0107	0.1037	**0.0020**
Merged two-three-four-five-source mixtures	Four-source mixtures	0.0489	0.0123	0.1120	**0.0030**
Merged two-three-four-five-source mixtures	Five-source mixtures	0.0659	0.0164	0.1253	**0.0040**

**Table 4:** Average RMSE results for “low-mixing-rate mixture” dataset (Mixing-rate range used for synthesizing mixtures is: [10^−3^ 10^−1^], Min: 10^−3^ Max: 10^−1^). Bold numbers indicate best performing algorithm.

Training Data	Test Data	NCLS	PLS	DBN	DR
Two-source mixtures	Two-source mixtures	0.4138 × 10^−3^	0.7989 × 10^−3^	4.2165 × 10^−3^	**0.3140 × 10^−3^**
Three-source mixtures	Three-source mixtures	1.6890 × 10^−3^	0.8518 × 10^−3^	3.5325 × 10^−3^	**0.4743 × 10^−3^**
Four-source mixtures	Four-source mixtures	0.6578 × 10^−3^	0.8792 × 10^−3^	3.3194 × 10^−3^	**0.5770 × 10^−3^**
Five-source mixtures	Five source mixtures	**0.6994 × 10^−3^**	0.9175 × 10^−3^	3.731 × 10^−3^	0.8196 × 10^−3^
Merged two-three-four-five-source mixtures	Two-source mixtures	0.4137 × 10^−3^	0.8541 × 10^−3^	3.1169 × 10^−3^	**0.3293 × 10^−3^**
Merged two-three-four-five-source mixtures	Three-source mixtures	1.6890 × 10^−3^	1.2341 × 10^−3^	3.5744 × 10^−3^	**0.5326 × 10^−3^**
Merged two-three-four-five-source mixtures	Four-source mixtures	0.6578 × 10^−3^	0.9574 × 10^−3^	3.7319 × 10^−3^	**0.5849 × 10^−3^**
Merged two-three-four-five-source mixtures	Five-source mixtures	**0.6994 × 10^−3^**	1.0262 × 10^−3^	4.2337 × 10^−3^	0.7652 × 10^−3^

**Table 5:** Robustness analysis results for “high-mixing-rate mixture” dataset (Mixing-rate range used for synthesizing mixtures is: [0.25 3.00], Min: 0.25 Max: 3.00). Bold numbers indicate best performing algorithm.

Training Data	Test Data	NCLS	PLS	DR
Two-source mixtures	Three-source mixtures	0.0317	**0.0114**	0.0141
Two-source mixtures	Four-source mixtures	0.0488	**0.0208**	0.0238
Two-source mixtures	Five-source mixtures	0.0658	**0.0321**	**0.0320**
Three-source mixtures	Two-source mixtures	0.0175	0.0093	**0.0029**
Four-source mixtures	Two-source mixtures	0.0175	0.0151	**0.0063**
Five-source mixtures	Two-source mixtures	0.0175	0.02269	**0.0145**

**Table 6:** Robustness analysis results for “low-mixing-rate mixture” dataset (Mixing-rate range used for synthesizing mixtures is: [10^−3^ 10^−1^], Min: 10^−3^ Max: 10^−1^). Bold numbers indicate best performing algorithm.

Training Data	Test Data	NCLS	PLS	DR
Two-source mixtures	Three-source mixtures	**0.0016**	0.0019	**0.0015**
Two-source mixtures	Four-source mixtures	**0.0006**	0.0008	0.0033
Two-source mixtures	Five-source mixtures	**0.0006**	0.0009	0.0049
Three-source mixtures	Two-source mixtures	**0.4137 × 10^−3^**	1.4944 × 10^−3^	0.7797 × 10^−3^
Four-source mixtures	Two-source mixtures	**0.4137 × 10^−3^**	0.8189 × 10^−3^	0.5300 × 10^−3^
Five-source mixtures	Two-source mixtures	**0.4137 × 10^−3^**	0.8084 × 10^−3^	0.9360 × 10^−3^

**Table 7:** Average RMSE results when foreground spectra are used for mixing-rate estimation in the “low-mixing-rate mixture” dataset (Mixing-rate range used for synthesizing mixtures is: [10^−3^ 10^−1^], Min: 10^−3^, Max: 10^−1^). Bold numbers indicate best performing algorithm.

Training Data	Test Data	NCLS	PLS	DR
Two-source mixtures	Two-source mixtures	0.0232	0.0006	**0.0003**
Three-source mixtures	Three-source mixtures	0.0268	0.0007	**0.0004**
Four-source mixtures	Four-source mixtures	0.0247	0.0007	**0.0005**
Five-source mixtures	Five-source mixtures	0.0250	0.00074	**0.00073**
Merged two-three-four-five-source mixtures	Two-source mixtures	0.0232	0.0007	**0.0003**
Merged two-three-four-five-source mixtures	Three-source mixtures	0.0268	0.0011	**0.0005**
Merged two-three-four-five-source mixtures	Four-source mixtures	0.0247	0.0009	**0.0005**
Merged two-three-four-five-source mixtures	Five-source mixtures	0.0250	0.0009	**0.0006**

**Table 8:** Average RMSE values of the test dataset with five methods using Source and Foreground spectra. Bold numbers indicate best performing algorithm.

Spectrum Type Used	NCLS	PLS	DR	LR	RFR
**Source**	0.0158	0.0119	**0.0028**	0.0128	0.0097
**Foreground**	0.0323	0.0115	**0.0024**	0.0123	0.0092

## Acronyms Used in This Paper

GADRAS	Gamma Detector Response and Analysis Software
NaI	Sodium Iodide
ML	Machine Learning
ROI	Region of Interest
NCLS	Non-negativity Constrained Least Square
PLS	Partial Least Square
DBN	Deep Belief Network
DR	Deep Regression
LR	Linear Regression
RFR	Random Forest Regression
ReLU	Rectified Linear Unit
RMSE	Root Mean Square Error
LSE	Least Square Error
GUI	Graphical User Interface
MD	Maryland

## References

[1] Durbin M., Kuntz A., Lintereur A. Machine Learning Applications for the Detection of Missing Radioactive Sources. Proceedings of the IEEE Nuclear Science Symposium and Medical Imaging Conference (NSS/MIC).

[2] Cordone G., Brooks R.R., Sen S., Rao N.S., Wu C.Q., Berry M.L., Grieme K.M. Regression for Radioactive Source Detection. Proceedings of the IEEE Nuclear Science Symposium and Medical Imaging Conference (NSS/MIC).

[3] Kim D., Yu D., Sawant A., Choe M.S., Choi E. First experimental observation of plasma breakdown for detection of radioactive material using a gyrotron in real-time. Proceedings of the Eighteenth International Vacuum Electronics Conference (IVEC).

[4] Eleon C., Battiston F., Bounaud M., Mosbah M.B., Passard C., Perot B. Study of Boron Coated Straws and mixed (10B/3He) detectors for passive neutron measurements of radioactive waste drums. Proceedings of the IEEE Nuclear Science Symposium and Medical Imaging Conference Proceedings (NSS/MIC).

[5] GADRAS. osti.gov/biblio/1166695-gadras-drf-user-manual.

[6] Olmos P., Diaz J., Perez J., Gómez P., Rodellar V., Aguayo P., Brú A., Garcia-Belmonte G., De Pablos J. (1991). A new approach to automatic radiation spectrum analysis. IEEE Trans. Nucl. Sci..

[7] Pilato V., Tola F., Martinez J., Huver M. (1999). Application of neural networks to quantitative spectrometry analysis. Nucl. Instrum. Methods Phys. Res. Sect. A Accel. Spectrometers Detect. Assoc. Equip..

[8] Yoshida E., Shizuma K., Endo S., Oka T. (2002). Application of neural networks for the analysis of gamma-ray spectra measured with a Ge spectrometer. Nucl. Instrum. Methods Phys. Res. Sect. A Accel. Spectrometers Detect. Assoc. Equip..

[9] Chen L., Wei Y.-X. (2009). Nuclide identification algorithm based on K–L transform and neural networks. Nucl. Instrum. Methods Phys. Res. Sect. A Accel. Spectrometers Detect. Assoc. Equip..

[10] Kamuda M., Stinnett J., Sullivan C.J. (2017). Automated Isotope Identification Algorithm Using Artificial Neural Networks. IEEE Trans. Nucl. Sci..

[11] Kamuda M. (2019). Automated Isotope Identification and Quantification Using Artificial Neural Networks. Ph.D. Thesis.

[12] Kwan C., Ayhan B., Chen G., Wang J., Ji B., Chang C.-I. (2006). A novel approach for spectral unmixing, classification, and concentration estimation of chemical and biological agents. IEEE Trans. Geosci. Remote Sens..

[13] Ayhan B., Kwan C., Galbacs G. Gold Fineness Determination Using LIBS Spectra with PLS and Spectral Unmixing Techniques. Proceedings of the 2nd International Conference on Applied and Theoretical Information Systems Research.

[14] Ayhan B., Kwan C. (2017). Application of deep belief network to land cover classification using hyperspectral images. International Symposium on Neural Networks.

[15] Keras Sequential Model. https://keras.io/guides/sequential_model/.

[16] Krizhevsky A., Sutskever I., Hinton G. (2012). Imagenet classification with deep convolutional neural networks. Adv. Neural Inf. Process. Syst..

[17] Ayhan B., Kwan C., Budavari B., Larkin J., Gribben D., Li B. (2020). Video Activity Recognition with Varying Rhythms. IEEE Access.

[18] Kwan C., Gribben D., Tran T. Tracking and Classification of Multiple Human Objects Directly in Compressive Measurement Domain for Low Quality Optical Videos. Proceedings of the IEEE 10th Annual Ubiquitous Computing, Electronics & Mobile Communication Conference (UEMCON).

[19] Rababaah A., Sharma D.K. (2015). Integration of two different signal processing techniques with artificial neural network for stock market forecasting. Acad. Inf. Manag. Sci. J..

[20] Ayhan B., Kwan C., Budavari B., Kwan L., Lu Y., Perez D., Li J., Skarlatos D., Vlachos M. (2020). Vegetation Detection Using Deep Learning and Conventional Methods. Remote Sens..

[21] Qu Y., Baghbaderani R.K., Qi H., Kwan C. (2021). Unsupervised Pansharpening Based on Self-Attention Mechanism. IEEE Trans. Geosci. Remote Sens..

[22] Top 20 Applications of Deep Learning in 2021 across Industries. https://www.mygreatlearning.com/blog/deep-learning-applications/#cars.

[23] Palm R.B. (2012). Prediction as a Candidate for Learning Deep Hierarchical Models of Data.

[24] Restricted Boltzman Machine. https://en.wikipedia.org/wiki/Restricted_Boltzmann_machine.

[25] Hinton G. (2010). A Practical Guide to Training Restricted Boltzmann Machines.

[26] Keras Dense Layer. https://keras.io/api/layers/core_layers/dense/.

[27] Kingma D.P., Ba J. (2014). Adam: A Method for Stochastic Optimization. arXiv.

[28] Freedman D.A. (2009). Statistical Models: Theory and Practice.

[29] Random Forest Regression. https://levelup.gitconnected.com/random-forest-regression-209c0f354c84.

[30] Decision Trees. https://scikit-learn.org/stable/modules/tree.html.

[31] CNS Global Incidents and Trafficking Database. http://nti.org/analysis/articles/cns-global-incidents-and-trafficking-database.

